# A Co-Culture Model of Fibroblasts and Adipose Tissue-Derived Stem Cells Reveals New Insights into Impaired Wound Healing After Radiotherapy

**DOI:** 10.3390/ijms161125935

**Published:** 2015-10-29

**Authors:** Frank Haubner, Dominique Muschter, Fabian Pohl, Stephan Schreml, Lukas Prantl, Holger G. Gassner

**Affiliations:** 1 Department of Otorhinolaryngology, Division of Facial Plastic Surgery, University Medical Center, Regensburg 93053, Germany; dominique.muschter@ukr.de (D.M.); holger.gassner@ukr.de (H.G.G.); 2 Department of Radiotherapy, University Medical Center, Regensburg 93053, Germany; fabian.pohl@ukr.de; 3 Department of Dermatology, University Medical Center, Regensburg 93053, Germany; stephan.schreml@ukr.de; 4 Department of Plastic and Reconstructive Surgery, University Medical Center, Regensburg 93053, Germany; lukas.prantl@ukr.de

**Keywords:** external radiation, fibroblasts, adipose derived stem cells, matrix metalloproteinases, tissue inhibitors of matrix metalloproteinases

## Abstract

External radiation seems to be associated with increased amounts of cytokines and other cellular modulators. Impaired microcirculation and fibrosis are examples of typical long term damage caused by radiotherapy. Adipose tissue-derived stem cells (ASC) are discussed to enhance wound healing, but their role in wounds due to radiotherapy is poorly understood. Normal human fibroblasts (NHF) and ASCs were co-cultured and external radiation with doses from 2–12 Gray (Gy) was delivered. Cell proliferation and mRNA levels of matrix metalloproteinases (*MMP1*, *MMP2* and *MMP13)* were determined 48 h after irradiation of the co-cultures by qPCR. Additionally, tissue inhibitors of matrix metalloproteinases (TIMP1, TIMP2) were determined by enzyme-linked immunosorbent assay (ELISA). There was a reduction of cell proliferation after external radiation in mono-cultures of NHFs and ASCs compared to controls without irradiation. The co-culture of ASCs and NHFs showed reduced impairment of cell proliferation after external radiation. Gene expression of *MMP1* and *MMP13* was reduced after external irradiation in NHF. *MMP2* expression of irradiated NHFs was increased. In the co-culture setting, *MMP1* and *MMP2* gene expression levels were upregulated. TIMP1 and TIMP2 protein expression was increased after irradiation in NHFs and their co-cultures with ASCs. ASCs seem to stimulate cell proliferation of NHFs and modulate relevant soluble mediators as well as proteinases after external radiation.

## 1. Introduction

Preoperative radiotherapy is a well-documented risk factor for postsurgical complications [1]. Patients suffering from persistent wounds after radiotherapy belong to the major challenges in head and neck surgery. The impaired healing of irradiated tissues results in an increased morbidity. Fibrotic tissue changes, pharyngo-cutaneous fistula, carotid artery exposure and occasional major vessel rupture may occur [2,3]. Extensive surgical efforts including microvascular reconstruction often fail in previously irradiated regions of the neck [4]. The diminished healing capacity of the irradiated tissues seems to be associated with fibrosis and decreased vascularity [4].

Wound healing represents a complex interaction of cells, cytokines, chemokines and various extracellular matrix proteins. Basically, wound repair comprises three major phases: inflammation, new tissue formation (cell proliferation, cell migration, neoangiogenesis), and tissue remodeling [5,6,7,8,9]. External radiation represents a serious damage to this well-organized network. Ongoing occurrences of inflammation and regeneration are the consequences [10].

In previous experiments with human dermal microvascular endothelial cells (HDMEC), we detected elevated concentrations of pro-inflammatory mediator molecules (cytokines/chemokines) in irradiated cell culture supernatants. [11].

Mesenchymal stem cells are suggested to support wound healing because they produce multiple growth factors and cytokines which are of major interest in wound healing processes [12,13]. The discovery that adipose-derived stem cells (ASC) are also multipotent progenitors of various cell types was an important step forward in the field of stem cell research. ASCs can be obtained easily by rather low invasive procedures such as liposuction [5].

The influence of ASCs on radiogenic wounds seems to be favorable according to reports in the area of breast reconstruction [14]. Larger clinical trials of head and neck patients which focus on the cellular interactions in radiogenic wounds are still missing.

As previously reported, we found a modulation of pro-inflammatory cytokines and adhesion molecules in an experimental co-culture setting of HDMECs and ASCs [15].

Besides endothelial cells, fibroblasts are key cells in wound healing. Dermal fibroblasts are responsible for the correct deposition and remodeling of collagen bundles. After external radiation, these cells seem to generate a disorganized network of collagen fibers. Dysregulation of matrix metalloproteinases (MMP) and their tissue inhibitors (TIMP) might be responsible for this effect. MMP and TIMP coordinate extracellular matrix production and seem to be essential during the inflammatory phase of wound healing [16,17,18]. That is why we evaluated the effects of external radiation on normal human fibroblasts (NHF) and the effects of adipose-derived stem cells (ASC) in a co-culture setting with respect to MMPs und TIMPs.

## 2. Results and Discussion

### 2.1. Results

#### 2.1.1. Effect of Irradiation on Cell Proliferation

**Figure 1:**
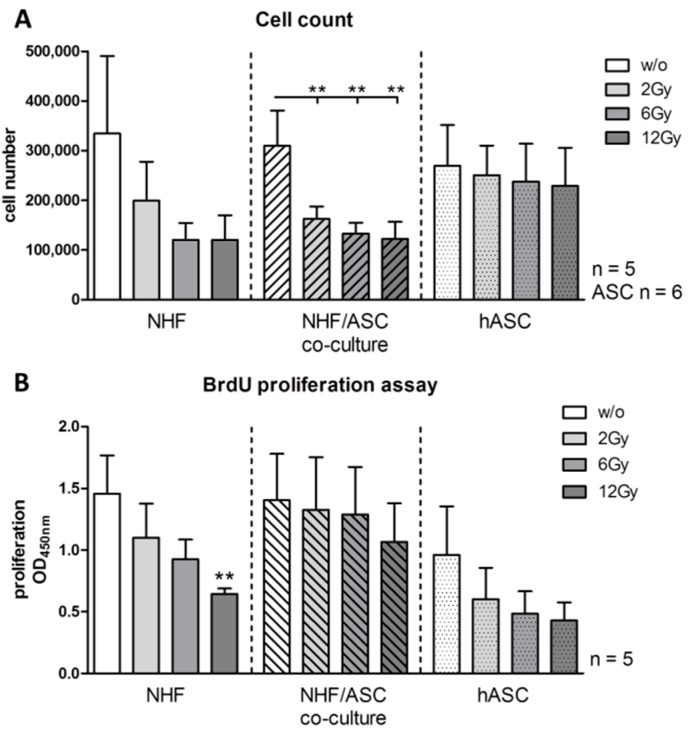
Absolute cell numbers (**A**) and cell proliferation (**B**) of viable adipose-derived stem cells (ASC), normal human fibroblasts (NHF) and the co-culture of ASC and NHF 48 h after irradiation with 2 to 12 Gy compared to unirradiated control cells determined by BrdU incorporation assay. Error bars represent standard error of the mean (*n* = 5, cell numbers ASC *n* = 6). ** *p* < 0.01.

First, to analyze the impact of external radiation on NHF, ASC and the respective co-culture, absolute cell numbers were determined 48 h after irradiation with 2, 6 and 12 Gy. In co-cultures of NHF and ASC, external radiation with 2, 6 and 12 Gy induced a significant decline in cell numbers compared with non-irradiated controls. NHF monocultures showed a similar, though non-significant, decline in absolute cell numbers whereas ASC monocultures seemed less affected by radiation (Figure 1A). Cell proliferation was determined by BrdU proliferation assays to further elucidate if changes in the proliferative capacity of cells causes the reduction in absolute cell numbers. External radiation resulted in a decreased cell proliferation of NHF. Cell proliferation was significantly reduced to 44% (*p* = 0.0079) after irradiation with 12 Gy in NHF monocultures. Cell proliferation of ASC monocultures was less affected by external radiation. There was no significant impact of external radiation on cell proliferation of co-cultures. (Figure 1B).

#### 2.1.2. Gene Expression of *MMP1* and *MMP2* in Normal Human Fibroblasts (NHF), Adipose-Derived Stem Cells (ASC) and Co-Culture of NHF and ASC

**Figure 2:**
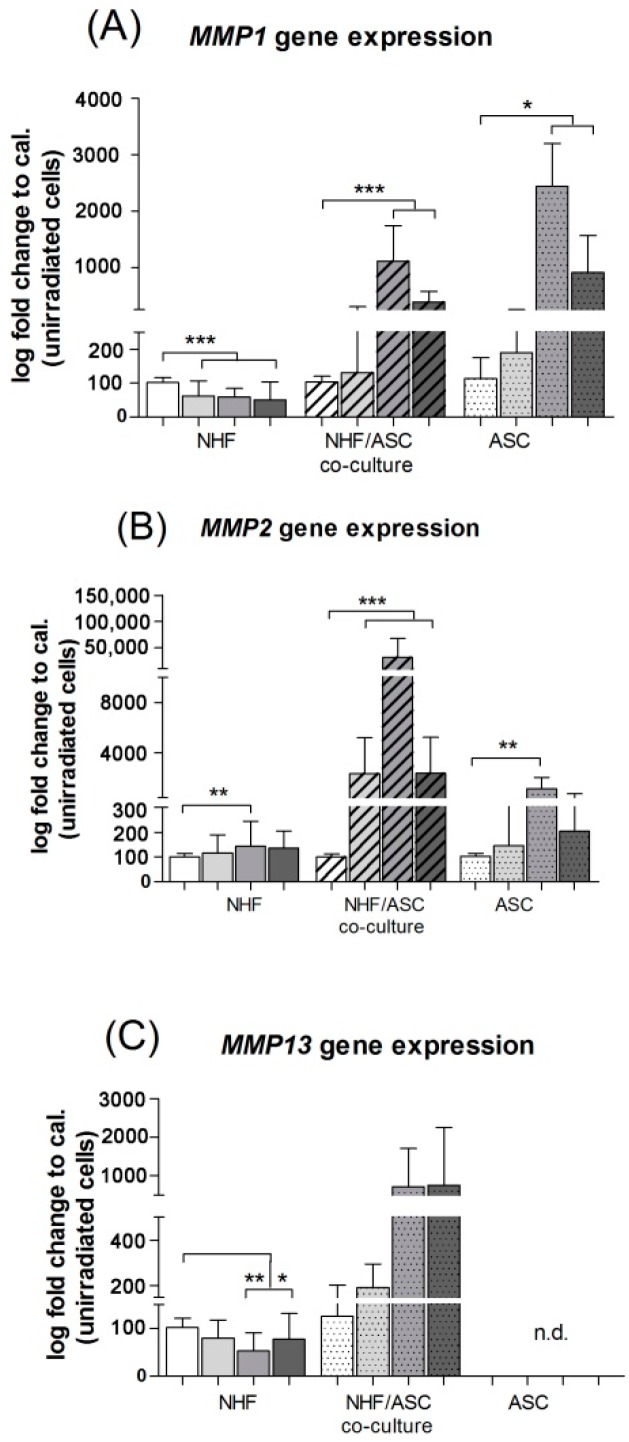
Relative expression of *MMP1* (**A**); *MMP2* (**B**) and *MMP13* (**C**) mRNA in normal human fibroblasts (NHF), adipose-derived stem cells (ASC) and the co-culture NHF/ASC 48 h after irradiation with 2–12 Gy as determined by PCR. Error bars represent standard error of the mean (*n* = 7). * *p* < 0.05, ** *p* < 0.01, *** *p* < 0.001. n.d. means not detectable.

NHF mono-cultures showed a significant reduction of *MMP1* gene expression in irradiated conditions with a maximum at 12 Gy where relative gene expression decreased from 100% ± 2.16% to 49.89% ± 7.55% (*p* < 0.0001). In contrast, NHF/ASC co-cultures revealed an increase of *MMP1* after irradiation and maximal gene expression was observed at 6 Gy. The relative gene expression rose from 100% ± 2.45% to 384.18% ± 27.01% in co-cultures (*p* < 0.0001) and 100% ± 8.83% to 912.46% ± 92.91% in ASC (*p* = 0.014) (Figure 2A).

Gene expression of *MMP2* was significantly increased from 100% ± 1.95% to 135.82% ± 9.92% (*p* = 0.0019) in NHF irradiated with 6 Gy compared to controls. Similarly, NHF/ASC co-cultures and monocultures of ASC showed a significantly elevated *MMP2* gene expression with a maximum after irradiation of 6 Gy. *MMP2* rose from 100% ± 1.74% to 2335.2% ± 412% in co-cultures (*p* = 0.0004) and from 100% ± 1.80% to 204.54% ± 71.81% in ASC (*p* = 0.0025) (Figure 2B).

Additionally, we analyzed the gene expression pattern of *MMP13.* In general, Ct values were high for NHF and NHF/ASC co-cultures, and no signal was detected for ASC monocultures. We revealed a significant down-regulation of *MMP13* in NHF mono-cultures irradiated with 6 and 12 Gy, also with the highest decrease at 6 Gy from 100% ± 2.81% to 77.39% ± 7.69% (*p* = 0.0025). Even though *MMP13* gene expression in NHF/ASC co-cultures tends to increase after irradiation, values were inconsistent and hence not significant (Figure 2C).

In summary, irradiation with 6 Gy mainly affected all three cultures with respect to gene expression of *MMP1*, *MMP2* and *MMP13*. In general, we revealed a significant decrease of *MMP1* and *MMP13* for NHF mono-cultures and a significant increase of *MMP1* and *MMP2* in NHF/ASC co-cultures and ASC monocultures (Figure 2A–C).

#### 2.1.3. TIMP1 and TIMP2 Protein Expression in NHF, ASC and Co-Culture of NHF and ASC

**Figure 3:**
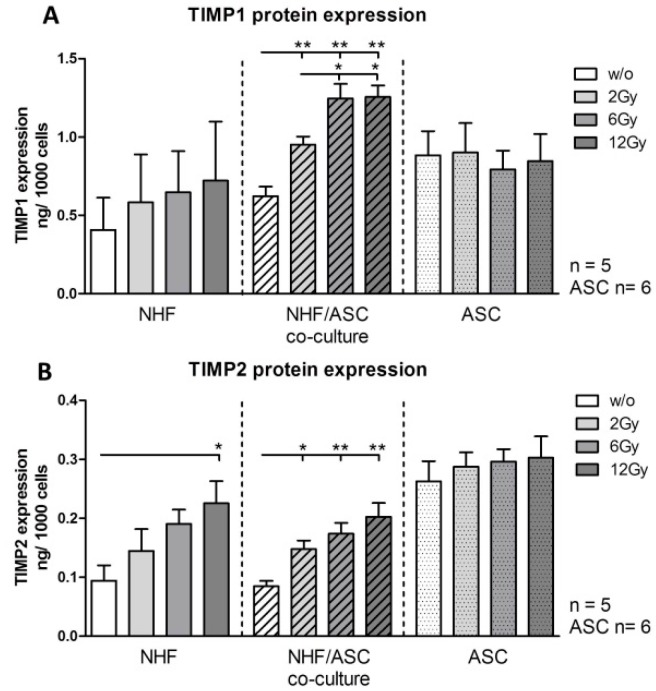
Protein expression of TIMP1 (**A**) and TIMP2 (**B**) in cell culture supernatants of NHF, ASC and co-cultured NHF/ASC 48 h after irradiation with 2–12 Gy compared to unirradiated (*w*/*o*) controls as determined by enzyme-linked immunosorbent assay (ELISA). Error bars represent standard error of the mean. NHF, co-cultures *n* = 5; ASC *n* = 6. * *p* < 0.05, ** *p* < 0.01.

NHF mono-cultures showed a significant increase of TIMP2 protein expression in irradiated conditions. Protein expression increased from 0.094 ng/1000 cells to 0.225 ng/1000 cells at 12 Gy (*p* = 0.0317). (Figure 3B) NHF/ASC co-cultures revealed an increase of TIMP1 and TIMP2 after irradiation. Maximal protein expression was observed at 12 Gy. The protein expression of co-cultures increased significantly from 0.622 ng/1000 cells for TIMP1 and 0.085 ng/1000 cells for TIMP2 to 1.256 ng/1000 cells (*p* = 0.0079) and 0.203 ng/1000 cells (*p* = 0.0079), respectively*. TIMP1 und TIMP2* protein expression of ASC monocultures were not affected by external radiation. (Figure 3A,B).

#### 2.1.4. TGF-β Protein Expression in NHF, ASC and Co-Culture of NHF and ASC

**Figure 4:**
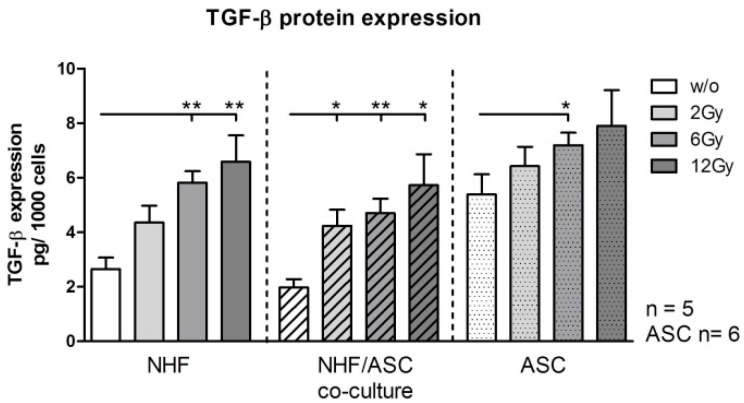
Protein expression of TGF-β in cell culture supernatants of NHF, ASC and co-cultured NHF/ASC 48 h after irradiation with 2–12 Gy compared to unirradiated controls as determined by ELISA. Error bars represent standard error of the mean. NHF, co-cultures *n* = 5; ASC *n* = 6. * *p* < 0.05, ** *p* < 0.01.

Expression of transforming growth factor (TGF)-β1 on the protein level was analyzed in the cell culture supernatants of ASC and NHF monocultures as well as of the respective co-cultures. External radiation significantly increased TGF-β protein concentration in the supernatants of NHF, ASC monocultures and their co-cultures. In the supernatants of NHF monocultures, the TGF-β concentrations increased from 2.64 pg/1000 cells in unirradiated cells to 5.81 (*p* = 0.0079) and 6.58 (*p* = 0.0079) pg/1000 cells upon exposure to doses of 6 and 12 Gy. Pre-radiation ASC expressed elevated amounts of TGF-β (5.38 pg/1000 cells) that were further significantly increased after radiation with 6 Gy to 7.18 pg/1000 cells (*p* = 0.0411). In co-culture settings, concentrations rose dose-dependently from 1.97 pg/1000 cells to 4.23 (2 Gy, *p* = 0.0159), 4.70 (6 Gy, *p* = 0.0079) and 5.72 (12 Gy, *p* = 0.0159) per 1000 cells, respectively (Figure 4).

### 2.2. Discussion

The treatment of head and neck cancer by radiotherapy is limited by serious side effects for the surrounding healthy tissue. External radiation often leads to poor wound healing and chronic ulcerations. Wound repair involves the interaction of dermal and epidermal cells, including fibroblasts, keratinocytes and endothelial cells [19].

Exposure to external radiation results in the development of fibrosis. Few therapeutic options exist for this pathology. Horton *et al.* [20] reported about the systemic infusion of bone marrow-derived mesenchymal stem cells after external radiation in mice. It was possible to reduce local inflammation and fibrosis by this intervention. Macrophage phenotype was even altered by bone marrow-derived stem cells.

Fibroblast proliferation is crucial to enable subsequent epidermal barrier repair. Fibroblasts stimulate the synthesis of extracellular matrix proteins, including type I and type III collagen, which are key components of granulation tissue [21,22].

Previous investigations of our group were focused on effects of external radiation on HDMECs [11,15]. The present study revealed a significant reduction of cell proliferation after external radiation of NHFs. Proliferation of ASC mono-cultures and co-cultures were less affected by irradiation. In our study, the results of the automatic cell counting system did not show complete apoptosis after external radiation. Decreased proliferation and an increased apoptotic cell ratio of fibroblasts occur after whole-body irradiation [23]. This effect of radiation was modulated in the co-culture setting with ASCs. With respect to the data of the present study, ASCs seem to protect and stimulate NHF proliferation because there was no significant decrease of cell proliferation after external radiation in the co-cultures.

Miller *et al.* [24] described specific histomorphological characteristics for radiation-induced skin damages. These include atrophy, dermal sclerosis and the loss of skin adnexal structures. Furthermore the hyalinization of vessel walls and atypical fibroblasts have been observed. There are two major theories concerning the formation of radiogenic ulcerations. One theory suggests microvascular compromise due to external radiation: The endothelial dysfunction may result in cell death and phenotype changes of endothelial cells. The other explanatory model is focused on fibroblast dysfunction because fibroblasts from irradiated tissues seem to be characterized by impaired proliferation rates [24]. These theories are supported by the experimental results of our present study on fibroblast proliferation and our previous results concerning endothelial dysfunction after external radiation [11].

In several studies, conditioned media initially incubated with ASCs promoted the proliferation and migration of dermal fibroblasts [25,26].

In an animal study by Lim *et al.* [27], ASCs significantly reduced the number of apoptotic salivary gland cells after external radiation. The authors suggested that ASCs might have the potential to protect against radiation-induced cell loss [27].

Protection and stimulation of cells which are crucial in wound healing might be one favorable effect of ASCs. The modulation of cytokines and factors that are responsible for extracellular matrix production represents an additional therapeutic option.

Comparable to the effect of external radiation on HDMECs, we found modulatory effects of ASCs concerning the IL-6 expression in NHFs [28]. IL-6 is a potent inducer of MMP1 and TIMP1 in dermal fibroblasts [29] and has also been reported to increase MMP13 expression in human chondrosarcoma cells by Tang *et al.* [30]. Another important factor involved in fibroblast differentiation and MMP expression in wound healing processes is TGF-β1 which has been found to be aberrantly expressed in radiation-induced fibrosis [31]. We could show that in NHF monocultures and in NHF co-cultures with ASC the concentrations of TGF increased with increasing doses of radiation irrespective of high doses of TGF-β1 originating from the serum contained in cell culture media.

In addition, we observed increased amounts of VEGF after external radiation of NHF and ASC mono-cultures in previous studies [28]. Irradiation and surgery are well known to induce hypoxia [32] which is a relevant factor in inducing VEGF transcription [33]. Furthermore, sufficient oxygenation is a crucial for cell growth and the synthesis of extracellular matrix proteins (ECM) in wound repair [34]. Former studies reported that cytokines might influence fibroblast proliferation and the synthesis of extracellular matrix proteins [35]. The link between those interactions are MMPs. MMPs are zinc-dependent neutral proteinases. They play important roles in angiogenesis, keratinocyte migration and tissue remodeling [36].

New data suggest that MMP1 and MMP9 control endothelial cell tube formation and morphogenic processes [37]. Furthermore, MMP1 is essential for the re-epithelialization of cutaneous wounds and the migration of fibroblasts [38]. MMP2 seems to have anti-inflammatory effects in acute wound healing and is suggested to reduce angiogenesis [38]. MMP13 expression is typically increased in chronic wounds and seems to promote endothelial cell migration [38]. These data underline the complex interactions of MMPs in tissue regeneration.

Our present study revealed a reduced gene expression of *MMP1* and *MMP13* and an increased expression of *MMP2* in irradiated NHF mono-cultures. After external radiation of ASC and NHF/ASC co-cultures, *MMP1* and *MMP2* gene expression were upregulated. MMP13 was not detectable in ASC and NHF/ASC co-cultures. Goessler *et al.* [39]., documented increased expression of the *MMP2*, *MMP12*, and *MMP13* in irradiated keratinocytes and fibroblasts Also Jourdan *et al.* [40], observed an elevated gene expression of MMP2 after skin radiation in rats in accordance to our results.

The finding of our present study, that *MMP1* gene expression, a suspected stimulator of neovascularization, is upregulated in the NHF/ASC co-culture setting supports the pro-angiogenic profile of ASC. Whether ASCs are suitable to influence MMP expression on the protein level has to be analyzed in further studies.

The balance between MMPs and their inhibitors (TIMP) has to be regulated precisely during the remodeling phase of wound healing [8].

Studies by Lee *et al.* [41] and Mueller *et al.* [42] observed elevated levels of TIMP1 in tissue samples from irradiated patients. TIMP1 and MMP1 belong to the ECM-remodeling-related molecules which are most affected by external radiation as shown by a microarray data [42]. Our present data revealed increased protein levels of TIMP1 (by trend) and TIMP2 after external radiation of NHF. These results support the findings of previous studies in the literature, which were performed on the gene expression level of TIMPs only [41,42]. The necessity to inhibit the actions of MMPs after external radiation was already emphasized by Riedel *et al.* [18]. That is why our finding that ASCs in co-culture with NHFs showed a significant increase in TIMP1/2 protein expression might be of further value. The exact interactions of MMPs and TIMPs in mesenchymal stem cells are still poorly understood. However, first *in vitro* studies have documented elevated TIMP synthesis from different mesenchymal stem cell sources [43].

However, it has to be considered that MMPs and TIMPs—like other enzymes—are pH-dependent. Extracellular pH-variations on wound surfaces dramatically alter MMP activity. Novel imaging tools for 2D luminescence imaging of pH appear particularly suitable to study 2D distribution patterns of pH-values. This would be suitable in order to plan *in vitro* studies on MMP function at the appropriate extracellular pH [44,45].

With respect to the findings of our present study, endogenous ASCs represent an important factor for wound repair. ASC are a valuable source of multipotent cells with characteristics comparable to bone marrow derived stem cells. Obvious advantages of ASC include the uncomplicated isolation process and their high concentration in fat tissue samples [46]. Further studies including non-irradiated ASCs are planned to understand their role as exogenous stem cell source.

In consideration of this limitation, ASCs seem to stimulate cell proliferation of NHFs and modulate relevant soluble mediators as well as proteinases after external radiation *in vitro*.

## 3. Experimental Section

### 3.1. Cell Culture

NHFs (Bio Whittaker Europe, Verviers, Belgium) were cultured in DMEM F-12 (Sigma-Aldrich, Munich, Germany) supplemented with 10% fetal bovine serum (FBS, Invitrogen, Darmstadt, Germany) and 1% penicillin/streptomycin (Sigma-Aldrich, Munich, Germany). NHF and were applied for experiments at passage 5. Culture incubator was set to 37 °C with 5% humidified CO_2_.

The isolation of ASC was performed as described previously by Gehmert *et al*. [47]. ASC were cultured in a medium with αMEM containing 20% FBS, 2 mM l-glutamine and 1% penicillin/streptomycin (Sigma, St. Louis, MO, USA). ASC were used for experiments at passages 5 and 6.

In summary, subcutaneous fat tissue, which was obtained during liposuction procedures was washed in phosphate-buffered saline and minced into pieces of <2 mm^3^. Serum-free MEM (1 mL/1 g tissue) and LiberaseBlendzyme 3 (Roche Diagnostics, Basel, Switzerland) (2 U/1 g tissue) were administered. Incubation was performed under continuous shaking at 37 °C for 45 min. The resulting solution was sequentially filtered through 100- and 40-μm filters (Fisher Scientific, Schwerte, Germany) and centrifuged at 450× *g* for 10 min. After discarding the supernatant, pelleted cells were washed two times with Hanks’ balanced salt solution (Cellgro, Manassas, VA, USA). Plastic-adherent passage 0 cells were cultured in vials (Greiner Bio-one, Frickenhausen, Germany) followed by daily washes in order to get rid of unattached cells and erythrocytes . After reaching 80% confluence in passage 0, the cells were seeded at a density of 3000 cells per cm^2^. The culture incubator was set at 37 °C with 5% carbon dioxide [47]. According to previous studies, ASCs maintain their differentiation capacity up to passage 15 [48].

The isolation process of ASC isolation was in accordance with the guidelines of the Declaration of Helsinki. ASC cultures were obtained and characterized by the Applied Stem Cell Research Center of the University of Regensburg. The process of stem cell harvesting was approved by the Institutional Review Board (IRB) of the University Medical Center Regensburg (“Human mesenchymal stem cells as a target for development of cell based regenerative therapies”, IRB #08/117).

### 3.2. Co-Culture of NHFs and ASCs

As control experiments, NHF or ASC were plated with densities of 40,000 cells per 6-well and were supplemented with 2 mL of the appropriate culture medium. In the direct co-culture (1:2) 20,000 cells of each NHF and ASC were mixed and seeded in a 6-well system and were cultured with 2 mL fibroblast growth medium. After 24 h medium was changed and supernatants were obtained at the end of culture time.

### 3.3. Cell-Radiation

Forty-eight hours after seeding, the 6-well plates were placed on the acceleration treatment couch. In order to compensate for the build-up effect, 2 cm thick plates of perspex were positioned above and below the tissue culture vials. As previously described by Pohl *et al.* [49], the external radiation was delivered via an anterior portal by a 6 MV linear accelerator (3 Gy/min; Primus, Siemens, Nuernberg, Germany) at room temperature. Dosimetric controls were performed to guarantee a homogenous dose distribution. The cells were treated with doses of 2, 6 and 12 Gy. Cells without radiation treatment provided the control values.

### 3.4. Cell-Harvesting

The supernatants of co-cultures were collected 48 h after external radiation, centrifuged 2 min at 13,000 rpm and stored at −20 °C until further analysis. Moncultures and co-cultures in the 6-wells were washed with PBS (phosphate-buffered saline, PAA laboratories, Pasching, Austria). Cell detachment was achieved by incubation with 500 µL Trypsin/EDTA (Promo-Cell; catalog number C-41000) for 5 min at 37 °C. Cell growth was identified with the Cedex XS cell counter system (Innovatis, Basel, Switzerland). Cells were pelletized and stored at −20 °C for further analysis.

### 3.5. Cell Proliferation Assay

Influence of external radiation on cell proliferation was analyzed using the colorimetric BrdU (5-bromo-2′-deoxyuridine, thymidine analog) Cell Proliferation ELISA from Roche (Basel, the Switzerland). Upon addition to the cell culture medium BrdU is constantly incorporated into newly synthesized DNA and its amount is proportional to the amount of newly formed DNA and hence allows comparison of proliferation rates of cells under varying conditions. Briefly, 2000 NHFs or ASCs were seeded as monocultures into the cavity of a 96-well plate. For co-cultures, 1000 cells of each NHFs and ASCs were used. Seventy-two hours after seeding, cells were irradiated with doses of 2, 6 and 12 Gy. Non-irradiated cells of each condition served as controls. Subsequently, after radiation, medium was exchanged for medium containing BrdU and cells were incubated for another 48 h. Afterwards, cells were processed following the manufacturer’s instructions, including the use of stop solution, and absorption was measured at 450 nm.

### 3.6. Enzyme-Linked Immunosorbent Assay (ELISA)

Soluble TIMP1 and TIMP2 protein production in the supernatants of monocultures and co-cultures, were analyzed by ELISA (DuoSet ELISA Development Systems; R&D Systems, Minneapolis, MN, USA). Initially, the cell culture supernatants were centrifuged at 5000 rpm for 5 min. The DuoSet kits human TIMP1 (DY970) and human TIMP2 (DY971) were used according to the manufacturer’s instructions, as well as the DuoSet ELISA Kit for human TGF-β1 (DY240) including an acid activation/neutralization step.

### 3.7. RNA Isolation and Reverse Transcriptase Quantitative Real-Time PCR

Total RNA was isolated using RNeasy Mini Kit (Qiagen, Hilden, Germany) according to manufacturer’s instructions and DNA was eliminated during transcription by the QuantiTect Reverse Transcription Kit (Qiagen, Hilden, Germany). Quantitative real-time PCR (qPCR) was performed in triplicate using cDNA concentrations that referred to 30 ng of RNA per reaction with Platinum SYBR Green qPCR SuperMix-UDG (Invitrogen, Carlsbad, CA, USA) on the MX3005 QPCR System (Stratagene, Agilent Technologies, Santa Clara, CA, USA). Relative quantification was evaluated with the MxPro QPCR software 4.1 (Stratagene, Agilent Technologies, Santa Clara, CA, USA) using human *GAPDH* (fw: 5′CTGACTTCAACAGCGACACC3′, rev: 5′CCCTGTTGCTGTAGCCAAAT3′) for normalization. The following primers were used for detection: human *MMP1* (fw: 5′ATGATATCTTTTGTCAGGGGAGAT3′, rev: 5′CCTGGTTGAAAAGCATGAGC3′), human *MMP2* (fw 5′GCCAATGGAGACTGTCTCAAGA3′, rev: 5′TTCTAAGGCAGCCAGCAGTGAA3′) and human *MMP13* (fw 5′CACCGGCAAAAGCCACTTTA3′; rev: 5′GACTGGTAATGGCATCAAGGGA3′). Results were calibrated on respective non-irradiated controls using the ∆∆*C*t method.

### 3.8. Statistical Analysis

Results are illustrated as mean ± standard error of the mean of independent experiments (different donors) performed in triplicate. We did Mann Whitney *U* tests (GraphPad Prism version 5.2, GraphPad Software, San Diego, CA, USA) for statistical analyses.

## 4. Conclusions

Adipose-derived stem cells seem to stimulate cell proliferation of human dermal fibroblasts and modulate relevant soluble mediators as well as proteinases after external radiation. The observed *in vitro* effects underline the wound healing promoting effects of ASC on irradiated tissues.
